# Stock market returns and oil price shocks: A CoVaR analysis based on dynamic vine copula models

**DOI:** 10.1007/s00181-021-02073-9

**Published:** 2021-06-01

**Authors:** Julia Kielmann, Hans Manner, Aleksey Min

**Affiliations:** 1 Department of Mathematics, Technical University of Munich, Munich, Germany; 2 Institute of Economics, University of Graz, Universitätsstr. 15/F4, 8010 Graz, Austria

**Keywords:** Oil prices, Risk management, Time-varying copula, D-vine copula, CoVaR, C12, C32, C52, C53

## Abstract

Crude oil plays a significant role in economic developments in the world. Understanding the relationship between oil price changes and stock market returns helps to improve portfolio strategies and risk positions. Kilian (Am Econ Rev 99(3): 1053–1069, 2009) proposes to decompose the oil price into three types of oil price shocks by using a structural vector autoregression model. This paper investigates the dynamic, nonlinear dependence and risk spillover effects between BRICS stock returns and the different types of oil price shocks using an appropriate multivariate and dynamic copula model. Risk is measured using the conditional value at risk, conditioning on one or more simultaneous oil and stock market shocks. For this purpose, a D-vine-based quantile regression model and the GAS copula model are combined. Our results show, inter alia, that the early stages of the Covid-19 crisis lead to increasing risk levels in the BRICS stock markets except for the Chinese one, which has recovered quickly and therefore shows no changes in the risk level.

## Introduction

Over the past decades, the emerging countries Brazil, Russia, India, China, and South Africa (BRICS) have grown rapidly and they have become more attractive for investors. In 1990, these five countries only generated about 11% of global gross domestic product (GDP), whereas in 2018 it was 32%. In the future, it is expected that this proportion will grow even more. This economic growth was accompanied by an increase of oil consumption. Therefore, it is important for global investors to investigate the relationship and risk spillover effects between the crude oil market and BRICS stock markets. This is particularly relevant in the light of increased financialization of the oil market, which is subject to speculation of financial investors; see Kilian and Murphy (2014); Kilian and Lee (2014). Consequently, there is danger of risk spillovers that investors and policy makers must be aware of.

Earlier studies focus on interactions between oil price shocks and the macroeconomy; see, e.g., Hamilton (1983), Bernanke (1983), Gilbert and Mork (1986), Hamilton (1988), Barsky and Kilian (2004), or Jones et al. (2004). Later, a considerable amount of literature considers the stock markets as the stock market usually is a good indicator for the economy in a country. For example, (Huang et al. (1996)) find no evidence for correlation between oil future returns and US stock returns. Sadorsky (1999), Jones and Kaul (1996) and Miller and Ratti (2009) suggest that oil prices have a negative influence on stock market returns. However, Sadorsky’s (2001) multifactor market model shows a positive relationship between oil price changes and stock price returns in the Canadian oil and gas industry. According to Basher and Sadorsky (2006) and Wang et al. (2013), there is a positive relationship for oil-exporting countries and a negative one for oil-importing countries.

However, these studies do not consider Kilian’s (2009) conclusion that not all oil price shocks are alike. He proposes an approach that decomposes the real price of oil into three components, i.e., three kinds of oil price shocks. Shocks to the current physical availability of crude oil, the *supply-side shocks*, occur for instance in the event of natural disasters. The *aggregate demand shocks* are shocks in the current demand for crude oil driven by fluctuations in the global business cycle, such as the global financial crisis. The *precautionary demand shocks* result from shifts in the precautionary demand for oil. Consequently, many subsequent studies follow the approach of Kilian (2009) and decompose the oil price shocks into the three different components by using a structural VAR (SVAR) model. Kilian and Park (2009) observe that oil price shocks caused by a global economic expansion have a positive effect on stock prices, whereas oil-demand shocks have a negative impact. Oil supply shocks seem to have a less significant influence on stock prices. Filis et al. (2011) investigate the time-varying correlation between stock market prices and oil prices using a DCC-GARCH-GJR approach in various oil-importing and oil-exporting countries. Their results show that there is a positive dependence between stock returns and oil demand-type shocks. Mokni (2020) recognizes by using a time-varying parameter regression model that stock returns react to the demand shocks more than to the supply shocks. Additionally, the effect of supply shocks is in general limited and negative, whereas aggregate demand shocks have a positive impact on stock returns. Basher et al. (2012) analyze the relationship between oil prices, exchange rates, and stock markets and find that positive oil supply shocks do not have a severe effect on the stock market prices. An unanticipated demand increase has a small positive impact on stock market prices. Moreover, many studies also show that the relationship between oil price shocks and stock market returns depends on the type of the economy, the political situation, and the importance of oil for the country. Due to the oil intensity in emerging countries, the economic impact of oil price shocks on emerging countries is generally higher than in industrialized countries (Li et al. 2020; Bouoiyour and Selmi 2016; Maghyereh 2006).

Many studies focus on linear relationship or even make the distributional assumption of normality. Moreover, they ignore the time variation of the relationship. In order to also capture nonlinear and asymmetric relationships, some studies employ the copula approach to explore the dependence between stock prices and oil price shocks. Aloui et al. (2013), Sukcharoen et al. (2014), and Li et al. (2020) use time-varying copula models to study the time-dependent relationship between oil prices and stock markets in various countries worldwide. However, these studies do not make a distinction between the different types of oil price shocks. A paper which combines the time-varying copula analysis and the SVAR-based decomposition of oil prices is the paper of Ji et al. (2018). This paper analyzes the dynamic dependence of BRICS countries’ stock returns and the three oil price shocks using the dynamic copula model by Patton (2006). Furthermore, a copula-based CoVaR approach is employed to study the risk spillover effects of oil price shocks on stock returns of the BRICS countries from the perspective of extreme market risks. The authors conclude that the dependence is generally time-varying and positive. The oil-specific demand shocks are the most important factor, and oil price shocks from the supply side do not significantly influence the stock market returns. The only exception is China, where the stock market is more sensitive to oil price increases resulting from oil supply shocks.

We consider a similar problem as Ji et al. (2018), studying the dependence properties between stock market returns in BRICS countries and the different types of oil prices shocks. However, we make the following contributions. First, we improve the bivariate modeling of time-varying copula dependence parameter by applying the generalized autoregressive score model (GAS), proposed by Creal et al. (2008). As shown in Manner and Reznikova (2012), the dynamic copula model by Patton (2006) is inferior to other specifications for time-varying copulas, so application of the GAS model is strictly preferable. Second, in order to be able to analyze also multivariate dependence structures, we extend the model by using a dynamic D-vine copula model, which combines the GAS model and the D-vine copula model, as proposed in Almeida et al. (2016). Third, we measure the spillover effects of multiple risk factors jointly using a copula-based CoVaR approach. As the computation of the copula-based CoVaR conditional on multiple variables exceeding certain risk levels is a non-standard problem, we propose using the D-vine-based quantile regression by Kraus and Czado (2017) to compute the required conditional quantile function. For this, we extend this approach for time-varying copulas. For a static vine copula a similar approach to computing the CoVaR has been proposed by Jiang et al. (2021). Finally, our data set spans the time period February 1996 to April 2020. With this data set, we can also derive some conclusions to early effects from Covid-19 crisis on the relationship between stock markets and the oil market.

This paper is organized as follows. Section 2 describes the econometric methodology. Section 3 presents the empirical analysis including the description and some further methodological issues. Finally, Sect. 4 concludes the paper.

## Methodology

We need an adequate model for the joint distribution of a vector time-series and follow the ideas of Almeida et al. (2016) combining the D-vine copula model with a generalized autoregressive score (GAS) model to allow for time-varying dependence parameters. This approach leads to the favorable situation that the joint distribution is separated into several bivariate time-varying copulas and its marginals. Then, we can apply a sequential maximum likelihood estimator (MLE) to estimate the required parameters.

We aim to model the joint distribution of a *d*-dimensional time series $\mathbf {r}_t = (r_{1,t},\dots ,r_{d,t})$ for $t =1, \dots , T$, where each $r_{i,t}$ follows a ARMA (*m*, *n*)-GARCH (*p*, *q*) model, i.e.,

2.1 $$\begin{aligned} r_{i,t}&= \mu _i + \sum _{j=1}^{m} \phi _j r_{i,t-j} + \epsilon _{i,t} + \sum _{j=1}^{n} \theta _j \epsilon _{i,t-j} = \mu _{i,t} + \epsilon _{i,t} \nonumber \\ \epsilon _{i,t}&= \sigma _{i,t} z_{i,t}, \quad z_{i,t} \sim F_{i} \text { } i.i.d.\nonumber \\ \sigma _{i,t}^2&= \omega _i + \sum _{j=1}^{p} \alpha _j \epsilon _{i,t-j}^2 + \sum _{j=1}^{q} \beta _j \sigma _{i,t-j}^2, \end{aligned}$$

where $\mu _{i,t} = E (r_{i,t} |\mathcal {F}_{t-1})$, $\sigma _{i,t}^2 = Var(r_{i,t} |\mathcal {F}_{t-1})$ and $F_{i}$ is the distribution function of the innovations with zero mean and unit variance. For the error distribution, we consider the normal and the skewed t-distribution by Hansen (1994). The best fitting ARMA-GARCH model is selected based on the BIC.

The joint distribution *F* of the standardized innovations $z_{i,t}$ can now be decomposed according to Sklar’s Theorem into its marginals $F_{1}, \dots F_d$ and its copula *C*:

$$\begin{aligned} F(z_{1,t}, \dots z_{d,t}) = C(F_1(z_{1,t}), \dots , F_d(z_{d,t})). \end{aligned}$$

The joint density is given by

2.2 $$\begin{aligned} f(z_{1,t}, \dots z_{d,t}) = c(F_1(z_{1,t}), \dots , F_d(z_{d,t})) \cdot \prod _{i=1}^{d} f_i(z_{i,t}), \end{aligned}$$

where *c* is the corresponding copula density. Estimation of the copula requires transforming the standardized innovations into uniformly distributed variables. For this purpose, we use the probability integral transformation and define the copula data as $u_{i,t} = F_i(z_{i,t},\hat{\varvec{\delta }}_i)$, where $\hat{\varvec{\delta }}_i$ denotes the estimated parameter vector for margin *i* and we define $\mathbf {u}_t = (u_{1,t}, \dots , u_{d,t})$. We assume that the parametric copula density *c* is time-varying, i.e., $\mathbf {u}_t \sim c(\varvec{u}_t| \varvec{\theta }_t, \mathcal {F}_{t-1}; \varvec{\gamma })$, where $\mathcal {F}_{t-1}$ is the information set available at time $t-1$, $\varvec{\theta }_t$ is the time-varying copula parameter, and $\varvec{\gamma }$ is the vector of time-independent parameters.

In Sect. 2.1, we introduce the GAS model, which allows modeling time-varying dependence in bivariate copula models. Section 2.2 introduces D-vine copulas, and in Sect. 2.3 we combine the time-varying GAS copulas and the D-vine model to obtain a flexible model allowing for high-dimensional and time-varying dependence. Section 2.4 reviews the value at risk and conditional value at risk, and Sect. 2.5 shows how D-vine-based quantile regressions can be used to compute the conditional value at risk in high-dimensional settings.

### Generalized autoregressive score copula model

The generalized autoregressive score (GAS) model was introduced in Creal et al. (2013). Consider a bivariate time series process $(u_{i,t}, u_{j,t})$ for $t =1, \dots , T$ and fixed $1\le i\ne j \le d$. Assume that its distribution is given by copula *c*, i.e.,

$$\begin{aligned} (u_{i,t}, u_{j,t}) \sim c(\cdot , \cdot ; \theta ^{ij}_t) \end{aligned}$$

with $\theta ^{ij}_t \in \Theta$ the time-varying parameter of the copula *c*. Without loss of generality, we assume that $\theta ^{ij}_t$ is a scalar for all *i*, *j* and *t*. To handle different copula families in a unified fashion, we parameterize the copulas with Kendall’s $\tau \in (-1,1)$, as for many bivariate copulas there is a one-to-one relationship between the copula parameter and Kendall’s $\tau$, i.e., there is a function *r* such that $\theta ^{ij}_t = r(\tau _t^{ij})$. We assume that $\tau _t^{ij}$ is driven by the process $\lambda _t^{ij} \in (-\infty ,\infty )$ through the inverse Fisher transform $\psi$,

$$\begin{aligned} \tau _t^{ij} = \frac{\mathrm{exp}(2\lambda _t^{ij})-1}{\mathrm{exp}(2\lambda _t^{ij})+1} =: \psi (\lambda _t^{ij}). \end{aligned}$$

Equivalently, we can express $\lambda _t^{ij} = \psi ^{-1} (\tau _t^{ij}) = 0.5 \cdot ln(\frac{1+\lambda _t^{ij}}{1-\lambda _t^{ij}})$. The GAS specification for $\lambda _t^{ij}$ is given by

$$\begin{aligned} \lambda _t^{i,j} = \omega _{ij} + \delta _{ij}s_{t-1}^{ij} + \phi _{ij}\lambda _{t-1}^{ij}, \end{aligned}$$

where $s_t^{ij}$ is the scaled score

$$\begin{aligned} s_t^{ij} = S_{ij,t}\nabla _{ij,t}, \end{aligned}$$

with the score

$$\begin{aligned} \nabla _{ij,t} = \frac{\partial \,ln\,c(u_{i,t}, u_{j,t}|\theta _t^{ij}, \mathcal {F}_{t}; \gamma _{ij})}{\partial \theta _t^{ij}} \end{aligned}$$

and $\gamma _{ij} = (\omega _{ij}, \phi _{ij}, \delta _{ij})$. The scaling factor $S_{ij,t}$ is defined as the square root of the inverse of the Fisher information.^1^

### D-vine models

The vine copula model enables us to construct multivariate copulas with a wide class of bivariate copula families as building blocks. The decomposition of the joint density into conditional pair copulas is not unique, and the number of possible constructions for a $d-$dimensional copula density is very large and equal to $$ \left( {\begin{array}{c}d\\ 2\end{array}}\right) \cdot (d-2)!\cdot 2^{\left( {\begin{array}{c}d\\ 2\end{array}}\right) }$$ (see Morales-Napoles et al. 2010). Therefore, Bedford and Cooke (2002) introduced a graphical model called regular vine (R-vine). The R-vine is a sequence of nested trees, which allows to organize and illustrate the needed pairs of variables and their corresponding sets of conditioning variables. D-vine copulas are a special case featuring a specific structure. The following statements are the basis for the vine copula theory. Let $(X_1, \dots , X_d)$ be a random vector with joint distribution *F* and density *f*, respectively. The density *f* can be decomposed recursively by

2.3 $$\begin{aligned} f(x_1, \dots , x_d) = \prod _{k=2}^{d} f(x_k|x_1, \dots , x_{k-1})\cdot f(x_1). \end{aligned}$$

Using Sklar’s Theorem, we can rewrite the conditional density $f(\cdot |\cdot )$ , e.g., for dimension $d=2$

$$\begin{aligned} f(x_1|x_2) = c_{12}(F_1(x_1), F_2(x_2)) \cdot f_1(x_1), \end{aligned}$$

where $c_{12}$ denotes the density of a bivariate copula. Similarly, for dimension $d=3$, we get

$$\begin{aligned} f(x_1|x_2,x_3) = c_{13|2}(F_{1|2}(x_1|x_2), F_{3|2}(x_3|x_2)|x_2) \cdot c_{12}(F_1(x_1), F_2(x_2)) \cdot f_1(x_1). \end{aligned}$$

For simplicity, we introduce for distinct indices $i,j,i_1, \dots , i_k$ with $i<j$ and $i_1<\dots <i_k$ the abbreviation

2.4 $$\begin{aligned} c_{i,j|D}:= c_{i,j|D}(F(x_i|x_D), F(x_j|x_D)), \end{aligned}$$

where $D:= \{i_1,\dots , i_k\}$ and $x_D:= (x_{i_1}, \dots , x_{i_k})$. With this notation, we can formulate the decomposition of conditional distribution of $(X_1,X_k)$ given $X_2 = x_2, \dots , X_{k-1} = x_{k-1}$

2.5 $$\begin{aligned} f(x_k|x_1, \dots , x_{k-1})= & {} c_{1,k|2:(k-1)} \cdot f(x_k|x_2,\dots , x_{k-1}) \nonumber \\= & {} \left[ \prod _{s=1}^{k-2} c_{s,k|(s+1):(k-1)}\right] \cdot c_{(k-1),k} \cdot f_k(x_k), \end{aligned}$$

where $r:s := (r,r+1, \dots , s)$ for integers *r* and *s* with $r<s$. Substituting (2.5) into (2.3) and replacing $s=i$, $k= i+j$ leads to

2.6 $$\begin{aligned} f(x_1, \dots , x_d) = \left[ \prod _{j=1}^{d-1} \prod _{i=1}^{d-j} c_{i,i+j|(i+1):(i+j-1)} \right] \cdot \left[ \prod _{k=1}^{d} f_k(x_k)\right] , \end{aligned}$$

where we drop the arguments of the copulas for simplicity. We see that the joint density can be separated into its marginal densities and several conditional pair copulas. In particular, if the marginal distributions of $X_k$ are uniform for all $k=1,\dots , d$, then the density in (2.6) is called a D-vine copula density and the corresponding distribution function is a D-vine copula distribution function. In general, $c_{i,i+j|(i+1):(i+j-1)} (\cdot ,\cdot )$ depends on the conditioning variables $x_{(i+1):(i+j-1)}$, so we make the common simplifying assumption that this dependence does not hold in our case. In our case, $c_{i,i+j|(i+1):(i+j-1)} (\cdot ,\cdot )$ only depends on the values of $F(X_i|x_{(i+1):(i+j-1)})$ and $F(X_{i+j}|x_{(i+1):(i+j-1)})$.

In order to compute the conditional distribution functions given in (2.4) for a D-vine copula, we use the following formula from Czado (2019). Let $i \in D$ and $D_{-i} := D \setminus \{i\}$, then

2.7 $$\begin{aligned} F(x_j|x_D) = h_{ji|D_{-i}}(F(x_j|x_{D_{-i}})|F(x_i|x_{D_{-i}})), \end{aligned}$$

where $h_{j|i}(u_j|u_i):= \frac{\partial C_{i,j}(u_i, u_j)}{\partial u_i}$ is the h-function associated with the pair copula $C_{ij}$.

The nodes in the D-vine tree represent the particular pairs of observations or pseudo observations obtained from previous trees and the edges represents the respective pair copula density with respective copula parameters. The pseudo-observations in the higher trees depend on the pair copulas in the lower trees. Based on the pseudo data $u_{j|D_{-i}}$ and $u_{i|D_{-i}}$ from the previous tree, the pseudo-observations for the next tree can be computed as

$$\begin{aligned} u_{j|D} = h_{ji|D_{-i}}(u_{j|D_{-i}}|u_{i|D_{-i}}; \theta _{ij|D}). \end{aligned}$$

Hence, the pseudo-data can be calculated recursively by starting with the copula data from the first tree and then using the bivariate copulas and pseudo-data from lower trees.

As an example, consider a D-vine copula density of dimension $d =5$. The joint copula density *c* can be written as

$$\begin{aligned} c(u_1,\dots , u_5) = c_{12} \cdot c_{23} \cdot c_{34} \cdot c_{45} \cdot c_{13|2} \cdot c_{24|3} \cdot c_{35|4} \cdot c_{14|23} \cdot c_{25|34} \cdot c_{15|234}. \end{aligned}$$

**Fig. 1:**
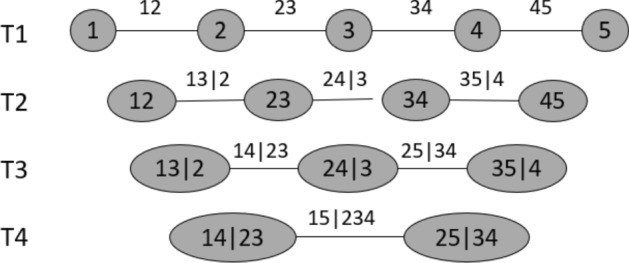
D-vine tree representation for $d=5$

The bivariate unconditional and conditional copula densities of the above factorization can be easily reconstructed from the corresponding D-vine tree shown in Fig. 1. For more details on vine trees and vine copulas, we refer to Czado (2019).

### D-vine-based multivariate dynamic copula models and estimation

Now, we introduce dynamics into the D-vine copula model by using the bivariate dynamic GAS copula as the pair copulas in a D-vine copula model. First, we assume that all conditional and unconditional pair copulas in (2.6) are bivariate parametric copulas. Further, we are given a time-varying copula parameter $\theta _t^{l(i,j)}$ for the bivariate copula density $c_{l(i,j)}(\cdot , \cdot ;\theta _t^{l(i,j)})$, where $l(i,j) := i,i+j|(i+1):(i+j-1)$ and $j=1, \dots , d-1$, $i=1, \dots , d-j$. Our dynamic parametric D-vine copula density is then given by

2.8 $$\begin{aligned} c(u_{1:d} \ ; \ \varvec{\theta _t}) := \prod _{j=1}^{d-1} \prod _{i=1}^{d-j} c_{l(i,j)} (F(u_i|u_{(i+1):(i+j-1)}),F(u_{i+j}|u_{(i+1):(i+j-1)});\theta _t^{l(i,j)}), \end{aligned}$$

where the parameter vector $\varvec{\theta _t}$ consists of the parameters of all pair copulas and we omit the parameters of the conditional marginal distributions.

The estimation of the model is done via inference function for margin (IFM) method by Joe and Xu (1996) as the logarithm of the joint density in (2.2) is a sum of marginal and copula log-likelihood functions. The idea of IFM is to maximize the log-likelihood separately. First, the marginal parameters are estimated separately and standardized residuals are formed. Then, we transform the standardized residuals with the parametric probability integral transformation in order to obtain copula data for the next step. According to (2.8), the copula density of a D-vine copula is a product of bivariate conditional copulas. Therefore, we can also estimate the copula parameters for each pair copula sequentially. We start in the first tree and utilize the estimation results from tree 1 to form pseudo copula data for the estimation of copula parameters in the second tree. We proceed with this approach until we have estimated all necessary pair copulas. According to Nagler et al. (2020), this multi-step estimator is consistent and asymptotically normal.

It is important to note that the order of variables in the first tree influences the estimation results and must be chosen with care. A common strategy is to choose the order of variables in the first tree such that neighboring variables have the highest Kendall’s Tau values among all possible orderings. We follow this procedure to determine the D-vine in our applications.

Density forecasts can be easily be computed from the suggested model, as the model is fully parametric and the time-varying parameters follow observation driven models. Hence, risk measures can be predicted in a straightforward fashion and the model can be used for portfolio optimization. We do not perform a prediction exercise in this paper due to the limited number of potential out-of-sample observations for a credible evaluation of these multivariate density forecasts^2^. However, the predictive performance of this model for weekly and daily stock (market) returns is studied in Almeida et al. (2016).

### Value at risk and conditional value at risk

The value at risk (VaR) is a common tool in financial risk management. It measures how much maximum loss an investor will suffer in a given time period and with a given probability. We distinguish between the upside and the downside risk. Given the log return $r_t$ of the underlying asset at time *t* and the confidence level $\alpha$, the VaR of a long financial position considers the lower tail of the return’s distribution and is defined as $P(r_t \le -VaR_{\alpha ,t}^{long}) = \alpha$. The VaR of a short financial position considers the upper tail of the return’s distribution and is defined as $P(r_t \ge VaR_{\alpha ,t}^{short}) =\alpha$. We define the market downside risk as $VaR_{\alpha ,t}^{D} = -VaR_{\alpha ,t}^{long}$ and the market upside risk as $VaR_{\alpha ,t}^{U} = VaR_{\alpha ,t}^{short}$. If the distribution $F_t$ of $r_t$ is continuous and strictly increasing, we can compute the VaR with help of the respective quantile function $F_t^{-1}$, i.e.,

$$\begin{aligned} VaR_{\alpha ,t}^{D} = F_t^{-1}(\alpha ) \text { , } VaR_{\alpha ,t}^{U} = F_t^{-1}(1-\alpha ). \end{aligned}$$

As the VaR only measures a single asset’s risk^3^, but we want to compute the systemic risk exposure of multiple assets and the risk spillover effects, we consider the conditional value at risk (CoVaR) as proposed by Adrian and Brunnermeier (2016). They define the CoVaR as the VaR of a financial system conditional on a given event. Consider an institution *i* and some event $C(X_j)$ with $j\ne i$. Then, the $CoVaR_\alpha$ is defined as the $\alpha$-quantile of the conditional probability of the financial system’s return $r_{i}$:

2.9 $$\begin{aligned} P(r_{i} \le CoVaR_{\alpha }^{i|C(X_j)} | C(X_j)) = \alpha . \end{aligned}$$

Here we define the event $C(X_j)$ as the situation when a market or various markets are in an extremal downside or upside risk situation with tail probability $\beta$. That means that these returns are equal to their respective downside or upside VaR. In the case of two observed markets, we define the downside CoVaR with tail probability $\alpha$ of market 1 at time t conditional on market 2 being in downside risk with tail probability $\beta$ as follows:

$$\begin{aligned} P(r_{1,t} \le CoVaR_{\alpha ,t}^{1|2,D} | r_{2,t} = VaR_{\beta ,t}^{2,D}) = \alpha . \end{aligned}$$

Similarly, the corresponding upside CoVaR with tail probability $\alpha$ can be defined as

$$\begin{aligned} P(r_{1,t} \ge CoVaR_{\alpha ,t}^{1|2,U} | r_{2,t} = VaR_{\beta ,t}^{2,U}) = \alpha . \end{aligned}$$

The CoVaR can be computed by solving this equation numerically. However, using the results from Hakwa et al. (2015), we can compute the CoVaR directly in our time-varying copula setting using the quantile function and the inverse *h*-functions:

2.10 $$\begin{aligned} CoVaR_{\alpha ,t}^{1|2,D}&= F_{1,t}^{-1} (h_{1|2} ^{-1}(\alpha ,F_{2,t}(VaR_{\beta ,t}^{2,D}); \theta _t^{1,2})) \nonumber \nonumber \\&= F_{1,t}^{-1} (h_{1|2} ^{-1}(\alpha ,\beta ;\theta _t^{1,2})),\nonumber \\ CoVaR_{\alpha ,t}^{1|2,U}&= F_{1,t}^{-1} (h_{1|2} ^{-1}(1-\alpha ,F_{2,t}(VaR_{\beta ,t}^{2,U}); \theta _t^{1,2})) \nonumber \\&= F_{1,t}^{-1} (h_{1|2} ^{-1}(1-\alpha ,1-\beta ;\theta _t^{1,2})), \end{aligned}$$

with $\theta _t^{1,2}$ the corresponding time-varying copula parameter of the copula $C_{12}$ associated to the returns $r_{1,t}$ and $r_{2,t}$.

### D-vine-based quantile regression

Equation (2.10) above allows us only to compute a bivariate copula-based CoVaR. In the empirical analysis we also want to stress more variables simultaneously, i.e., we want to consider more general events $C(X_j)$ that involve extreme events of various variables. To extend the copula-based CoVaR approach to such a multivariate setting, we make use of the D-vine-based quantile regression proposed by Kraus and Czado (2017). The quantile regression means prediction of conditional quantiles. This is exactly how the CoVaR is defined.

Let us assume that we want to predict the quantile of the return *Y* of asset 1 (response variable) given the returns $(X_1, \dots , X_d)$ of assets $2, \dots , d+1$, $d \ge 1$ (predictor variables), where $Y \sim F_Y$ and $X_j \sim F_j$, $j= 1, \dots d$. We are interested in the conditional quantile function

$$\begin{aligned} q_{\alpha }(x_1, \dots , x_d) := F_{Y|X_1, \dots X_d}^{-1}(\alpha |x_1, \dots , x_d), \qquad \alpha \in (0,1). \end{aligned}$$

We can express the joint conditional distribution function of *Y* as follows:

$$\begin{aligned} F_{Y|X_1, \dots X_d}(y|x_1, \dots , x_d)&= P(Y \le y| X_1 = x_1, \dots , X_d = x_d) \\&= P\left( F_Y(Y) \le v | F_1(X_1) = u_1, \dots , F_d(X_d) = u_d\right) \\&= C_{V|U_1, \dots U_d}(v|u_1, \dots , u_d), \end{aligned}$$

where $V := F_Y(Y)$, $U_j := F_j(X_j)$, $v := F_Y (y)$ and $u_j := F_j(x_j)$, $j = 1, \dots , d$. Inversion yields

2.11 $$\begin{aligned} F_{Y|X_1, \dots X_d}^{-1}(\alpha |x_1, \dots , x_d) = F_Y^{-1}\left( C_{V|U_1, \dots U_d}^{-1}(\alpha |u_1, \dots , u_d)\right) . \end{aligned}$$

Hence, we can express the conditional quantile function in terms of the inverse marginal distribution function $F^{-1}_Y$ of the response *Y* and the conditional copula quantile function $C_{V|U_1, \dots , U_d}^{-1}$. To get an estimate of the conditional quantile function, we have to first obtain the estimates of the marginals $\hat{F}_Y$ and $\hat{F}_j$, $j =1,\dots , d$, then the estimates of the copula $\hat{C}_{V|U_1,\dots ,U_d}$ and finally use the expression:

2.12 $$\begin{aligned} \hat{q}_{\alpha }(x_1,\dots , x_d) := \hat{F}_Y^{-1}\left( \hat{C}_{V|U_1, \dots U_d}^{-1}(\alpha |\hat{u}_1, \dots , \hat{u}_d)\right) , \end{aligned}$$

where $$\hat{u}_j:=\hat{F}_j(x_j)$$, $$j=1,...,d$$.

The difficulty lies now in the estimation of the multivariate copula $C_{V|U_1,\dots ,U_d}$. The solution is to fit a D-vine copula to $(V,U_1, \dots , U_d)$ such that V, as the response variable, is the first node in the first tree. The predictor variables are ordered such that the neighboring variables have the highest Kendall’s Tau values among all possible orderings. Finally, we can express the conditional copula function in terms of nested h-functions by using Eq. (2.7) as shown by Kraus and Czado (2017). We can extend the above equations for time-varying copulas. We illustrate this in a 4-dimensional example by letting the copula parameter be time-varying. The example is inspired by the example in Kraus and Czado (2017).

#### Example 1

Assume we want to compute the conditional distribution of the variable $V_1$ conditioned on the variables $V_2,V_3,V_4$. Moreover, assume that the optimal D-vine order is: $V_1-V_2-V_3-V_4$. Then, using Eq. (2.7), we can express the conditional distribution of $V_1$ given $(V_2,V_3,V_4)$ as follows:

$$\begin{aligned}&C_{V_1|V_2,V_3,V_4}(v_1|v_2,v_3,v_4; \varvec{\theta _t}) \\&\quad = h_{V_1|V_4;V_2,V_3}\left( C_{V_1|V_2,V_3}(v_1|v_2,v_3; \theta _t^{13|2})|C_{V_4|V_2,V_3}(v_4|v_2,v_3; \theta _t^{24|3}); \theta _t^{14|23}\right) \\&\quad = h_{V_1|V_4;V_2,V_3}(h_{V_1|V_3;V_2}\left[ C_{V_1|V_2}(v_1|v_2;\theta _t^{12})|C_{V_3|V_2}(v_3|v_2;\theta _t^{23}); \theta _t^{13|2}\right] |\\&h_{V_4|V_2;V_3} \left[ C_{V_4|V_3}(v_4|v_3;\theta _t^{34})|C_{V_2|V_3}(v_2|v_3;\theta _t^{23});\theta _t^{24|3}\right] ; \theta _t^{14|23}) \\&\quad = h_{V_1|V_4;V_2,V_3}( h_{V_1|V_3;V_2}\left[ h_{V_1|V_2}(v_1|v_2;\theta _t^{12})|h_{V_3|V_2}(v_3|v_2;\theta _t^{23});\theta _t^{13|2}\right] |\\&h_{V_4|V_2;V_3}\left[ h_{V_4|V_3}(v_4|v_3;\theta _t^{34})|h_{V_2|V_3}(v_2|v_3;\theta _t^{23});\theta _t^{24|3}\right] ;\theta _t^{14|23}) \end{aligned}$$

with $\varvec{\theta _t} = (\theta _t^{12}, \theta _t^{23},\theta _t^{34}, \theta _t^{13|2}, \theta _t^{24|3}, \theta _t^{14|32})$ the time-varying dependence parameter for each pair copula of the D-vine copula density.

By inverting the above expression, we get the conditional quantile function:

$$\begin{aligned}&C^{-1}_{V_1|V_2,V_3,V_4}(\alpha |v_2,v_3,v_4;\varvec{\theta _t}) \\&\quad =h^{-1}_{V_1|V_2}\{h^{-1}_{V_1|V_3;V_2}[h^{-1}_{V_1|V_4;V_2,V_3}\left( \alpha |h_{V_4|V_2;V_3}(h_{V_4|V_3}(v_4|v_3;\theta _t^{34})| \right. \\&\quad \left. h_{V_2|V_3}(v_2|v_3;\theta _t^{23});\theta _t^{24|3});\theta _t^{14|23}\right) |h_{V_3|V_2}(v_3|v_2;\theta _t^{23})];\theta _t^{13|2} |v_2; \theta _t^{12}\}. \end{aligned}$$

Using Eq. (2.12), we get a closed expression for the conditional quantile function for dimension $n=4$:

$$\begin{aligned} q_{\alpha }(v_2, v_3, v_4) = F_{V_1}^{-1}\left( C^{-1}_{V_1|V_2,V_3,V_4}(\alpha |v_2,v_3,v_4;\varvec{\theta _t})\right) . \end{aligned}$$

For the implementation of the time-varying D-vine-based quantile regression, we adopt code from the R package *vinereg*.

## Empirical analysis

Our aim is to investigate possible risk spillover effects between BRICS stock returns and different types of oil price shocks. In Sect. 3.1, we apply the structural VAR to decompose the oil price into three types of oil price shocks. Sect. 3.2 presents the data and preliminary analysis. In Sect. 3.3, we utilize a time-varying copula approach to model the bivariate dependence between BRICS stock returns and oil price shocks. In Sect. 3.4 we conduct a multivariate risk spillover analysis by using dynamic D-vine models.

### Decomposing oil prices

Kilian (2009) assumes that the real price of oil can be decomposed into three oil price shocks, the oil supply shocks, the aggregate demand shocks, and the precautionary demand shocks. In order to extract these effects from the monthly crude oil price, he utilizes a structural vector autoregressive (SVAR) model with lag order 24. The SVAR representation is given by

3.1 $$\begin{aligned} \mathcal {A} \mathbf {y}_t = \varvec{\nu } + \sum _{i=1}^{24} A_i \mathbf {y}_{t-i} + \varvec{\epsilon _t}, \end{aligned}$$

where $\mathbf {y}_t = (s_t, g_t, p_t)'$, $\varvec{\epsilon _t} = (\epsilon _t^{SS}, \epsilon _t^{DS}, \epsilon _t^{OS})'$ with $s_t-$ the percent change (log difference) in global crude oil production, $g_t-$ an index of real economic activity, and $p_t = 100\ln \left( \frac{np_t}{CPI_t/100}\right) -$ the real oil price expressed logarithmically. Here, $np_t$ refers to the nominal oil prices and $CPI_t$ to the U.S. consumer price index.

We follow the identification strategy in Kilian (2009) assuming a lower triangular form of $\mathcal {A}$ and the variable ordering given above. He argues that supply cannot be expected to respond to demand shocks in the same month as oil-producers need some time to react to changes in demand. Furthermore, it appears reasonable that aggregate demand does not respond immediately to oil market-specific shocks. Estimation is based on the reduced form representation. The structural shocks $\epsilon _t$ are obtained through the Cholesky decomposition of the covariance matrix of the reduced-form residuals.

The (structural) *oil price shocks* are defined as follows:

- $\epsilon _t^{SS}$ (crude oil supply shocks )—unpredictable innovations in global oil production,
- $\epsilon _t^{DS}$ (aggregate demand shocks)—unpredictable innovations in global real economic activity that cannot be explained based on crude oil supply shocks,
- $\epsilon _t^{OS}$ (oil-specific demand shocks)—unpredictable innovations in real oil price that cannot be explained based on oil supply shocks and aggregate demand shocks.

The oil price decomposition is computed as the cumulative effect of oil supply shock $p_t^{SS}$, of aggregate demand shock $p_t^{DS}$, and of oil-specific demand shock $p_t^{OS}$ as follows:

3.2 $$\begin{aligned} p_t^{\mathrm{SS}} = \sum _{q=0}^{t-1} \frac{\partial p_t}{\partial \epsilon _{t-q}^{\mathrm{SS}}} \hat{\epsilon }_{t-q}^{\mathrm{SS}}, \quad p_t^{\mathrm{DS}} = \sum _{q=0}^{t-1} \frac{\partial p_t}{\partial \epsilon _{t-q}^{\mathrm{DS}}} \hat{\epsilon }_{t-q}^{\mathrm{DS}}, \quad p_t^{\mathrm{OS}} = \sum _{q=0}^{t-1} \frac{\partial p_t}{\partial \epsilon _{t-q}^{\mathrm{OS}}} \hat{\epsilon }_{t-q}^{\mathrm{OS}}, \end{aligned}$$

where $p_t = c + p_t^{\mathrm{SS}} + p_t^{\mathrm{DS}} + p_t^{\mathrm{OS}}$ with a constant *c*, and $p_t^{\mathrm{SS}}, p_t^{\mathrm{DS}}, p_t^{\mathrm{OS}}$ the components of real oil prices, i.e., *oil supply shocks*, *aggregate demand shocks*, and *oil-specific demand shocks*.

Our analysis is based on the first differences of the oil price components $\Delta p_t^{\mathrm{SS}}$, $\Delta p_t^{\mathrm{DS}}$, and $\Delta p_t^{\mathrm{OS}}$, which we consider as oil price shocks.

### Data and preliminary analysis

In order to extract the three oil price shocks, we took monthly data from February 1994 to April 2020, i.e., 327 observations, of the following three variables : (1) World oil production as a proxy of *world oil supply* $s_t$ from the U.S. Energy Information Administration (EIA), (2) the Kilian index as the *global economic activity index* $g_t$ which we collected from the personal website of Lutz Kilian^4^, and (3) United States crude oil imported acquisition cost by refiners (collected from the EIA) deflated with the U.S. Consumer Price Index (collected from U.S. Bureau of Labor Statistics) as the *real price of oil* $p_t$.

The Kilian index is constructed from an equally weighted index of the percentage growth rates, obtained from a panel of single voyage bulk dry cargo ocean shipping freight rates measured in dollars per metric ton. The construction of the index controls for fixed effects associated with shipping routes, ship sizes, and types of cargo. We refer to Kilian (2009) for more details.

**Fig. 2:**
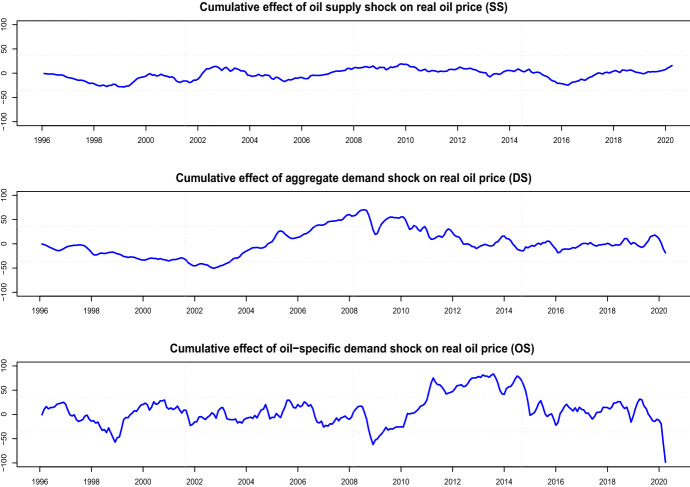
Historical decomposition of real oil prices

Using these variables, the SVAR(24) is estimated as described in Sect. 3.1. Figure 2 presents the evolution of the three components of real oil price, $p_t^{SS}, p_t^{DS}, p_t^{OS}$, defined in (3.2). We observe that oil prices are driven by different factors in different time periods. The supply shocks have a comparably small effect on the oil price and are relatively constant. The most volatile effect on the oil price is due to the oil-specific demand shocks. The drop and the subsequent increase of oil price during the Asian financial crisis in 1998/99 are contributed solely to the oil-specific demand shocks. The strong decrease of oil price due to the global financial crisis in 2008 has resulted especially from oil-specific demand shocks, but also from aggregate demand shocks. The same is valid for the Covid-19 crisis in 2020. Thereby, we can confirm the claim of Kilian and Park (2009) and Mokni (2020) that the oil price is mainly influenced by the demand side and there is little influence by the supply side. For the further analysis we use the oil price shocks defined as $\Delta p_t^{\mathrm{SS}}$, $\Delta p_t^{\mathrm{DS}}$, and $\Delta p_t^{\mathrm{OS}}$.

Moreover, as we want to analyze the dependence between different oil price shocks and BRICS stock returns, we collected monthly BRICS stock returns from investing.com, yahoo finance and the official website of BSE India. We summarize the used price indices in Table 1. The choice of the price index for a particular country was mainly driven by the availability of the data and the importance of the index.

**Table 1:** Price indices for each of the BRICS countries

Country	Price index name
Brazil	Bovespa (BVSP) Index
Russia	RTS (IRTS) Index
India	S&P BSE Sensex (BSESN) Index
China	Shanghai Composite (SSEC) Index
South Africa	FTSE/JSE All Share (JALSH) Index

**Fig. 3:**
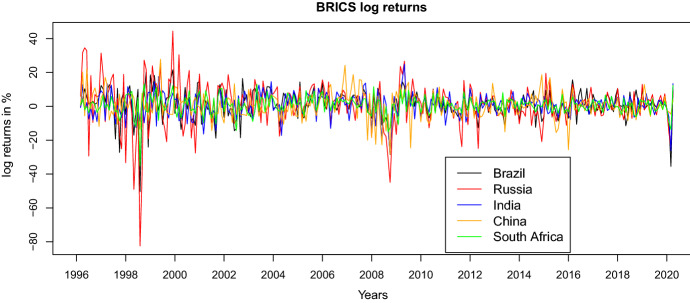
Stock market returns of BRICS countries

Figure 3 depicts the evolution of the log returns of the BRICS countries’ stock market indices for the period of February 1996 till April 2020. We notice a quite similar development of the stock returns. Russia and Brazil exhibit the most volatile log returns, whereas South Africa has the lowest volatility. Overall, we observe three periods with exceptionally large losses. The first is the Asian crisis in 1998, the second the global financial crisis in 2008, and the last the Covid-19 crisis in 2020.

Table 2 presents the summary statistics of the log returns in each of the BRICS countries and the three oil price shocks. The table shows that the monthly means of all stock returns of the BRICS are positive and Brazil has the highest monthly mean return or $0.961\%$ and China the lowest monthly mean return or $0.567\%$. Russia’s log returns have the largest and South Africa’s the lowest standard deviation. The skewness is negative for all countries, implying that the log returns are skewed to the left. Furthermore, the kurtoses are all positive, indicating a heavy tailed distribution of the log returns. The significant results of the Jarque-Bera statistics show evidence that BRICS returns exhibit a non-normal distribution. The Ljung-Box test with lag order six indicates that there exists autocorrelation for the Russian and Chinese return series. The Ljung-Box of squared residuals and the Lagrange multiplier test statistics with lag order six indicate that all BRICS log returns exhibit heteroskedasticity and ARCH effects.

The summary statistics of the oil price shocks reveal that the oil supply shocks have positive mean, while the other two oil shocks have negative mean. The oil-specific demand shocks have the largest standard deviation of 8.292 among all oil shock types. Besides, the test statistics show that all shocks are serially correlated. The Jarque-Bera statistic indicates a normal distribution for the supply shocks. Additionally, the aggregate demand shocks and the oil-specific demand shocks exhibit conditional heteroskedasticity.

**Table 2:** Summary statistics for oil price shocks and log returns of BRICS countries

	Mean	Median	Max.	Min.	Sd	Skew.	Kurt.
*BRICS*							
BR	0.961	1.258	21.546	−50.341	8.522	−1.315	5.387
RU	0.951	1.472	44.456	−82.457	13.270	−1.122	6.184
IN	0.792	0.971	24.885	−27.299	6.906	−0.464	1.490
CN	0.567	0.635	27.805	−28.278	7.796	−0.179	1.806
ZA	0.726	0.950	13.195	−35.134	5.384	−1.111	6.227
*OIL*							
SS	0.057	−0.013	7.065	−6.774	2.256	−0.094	3.123
DS	−0.062	0.274	11.262	−16.233	3.786	−0.770	5.204
OS	−0.337	1.062	19.258	−41.063	8.292	−0.946	5.898

	JB test		LB test(6)		LB$^2$-test(6)	ARCH-LM test(6)	
*BRICS*							
BR	442.831 $$^{***}$$		8.557		13.492 $$^{**}$$	19.485 $$^{***}$$	
RU	533.122 $$^{***}$$		20.507 $$^{***}$$		55.569 $$^{***} $$	77.331 $$^{***}$$	
IN	38.480 $$^{***}$$		1.419		15.602 $$^{**}$$	35.923 $$^{***}$$	
CN	42.454 $$^{***}$$		16.125 $$^{**}$$		21.651 $$^{***}$$	43.710$$^{***}$$	
ZA	538.481 $$^{***}$$		10.090		14.618 $$^{**}$$	20.930 $$^{***}$$	
*OIL*							
SS	0.688		83.407 $$^{***}$$		9.062	11.469 $$^{*}$$	
DS	89.633 $$^{***}$$		135.726 $$^{***}$$		121.823 $$^{***} $$	57.778 $$^{***}$$	
OS	148.064 $$^{***}$$		57.984 $$^{***}$$		60.635 $$^{***}$$	22.189 $$^{***}$$	

*JB* *denotes the Jarque-Bera normality test*, *LB* *the Ljung-Box test*, *LB*$$^2$$ *the Ljung-Box test for the squared series*. $$*$$, $$**$$, *and* $$***$$ *denote significance level at* $$10\%$$, $$5\%$$ *and* $$1\%$$, *respectively*.

In view of the descriptive statistics, it is reasonable to apply ARMA(*m*, *n*)-GARCH(*p*, *q*) models for the marginal distributions of the returns $r_{i,t}$ of country *i* at time point *t*, as defined in (2.1). For the conditional mean, we choose simple ARMA models with lag length minimizing the Bayesian information criterion (BIC). The conditional variance of the residuals is modeled using a GARCH(1, 1) model with either normal or skewed t distribution, again depending on the minimum BIC.

According to the BIC, we apply an ARMA(1, 0)-GARCH(1, 1) model with a normal distribution as conditional distribution of the standardized residuals for the two oil demand shocks and an ARMA(1, 1)-GARCH(1, 1) model with a normal distribution for the oil supply shock. For Brazil, Russia, and India, we employ an ARMA(0, 0)-GARCH(1, 1) model with skewed t innovations and for China and South Africa, we employ an ARMA(0, 0)-GARCH(1, 1) model with normal distribution. The estimated parameters (and their standard errors) of the marginal distributions are given in Table 3. Most coefficients are significant at the $5\%$-level. Moreover, we ensure with a Ljung-Box test that the residuals and squared residuals show no autocorrelation.

**Table 3:** ARMA-GARCH estimation results

	${\mu }$	${\phi _1}$	${\theta _1}$	${\omega }$	${\alpha _1}$	${\beta _1}$	Skew	Shape
SS	0.046	-0.347$$^{**}$$	0.723$$^{***}$$	0.230	0.045	0.905$$^{***}$$	–	–
	(0.211)	(0.116)	(0.085)	(0.161)	(0.026)	(0.046)	–	–
DS	0.110	0.662$$^{***}$$	–	0.212	0.211 $$^{**}$$	0.785$$^{***}$$	–	–
	(0.130)	(0.047)	–	(0.157)	(0.071)	(0.069)	–	–
OS	0.296	0.303$$^{***}$$	–	37.575$$^{***}$$	0.365$$^{**}$$	0.000	–	–
	(0.400)	(0.063)	–	(5.059)	(0.127)	(0.000)	–	–
BR	0.879$$^{*}$$	–	–	2.170	0.062	0.908$$^{***}$$	0.725$$^{***}$$	8.230$$^{*}$$
	(0.444)	–	–	(2.249)	(0.047)	(0.069)	(0.083)	(3.312)
RU	0.997	–	–	6.210$$^{*}$$	0.216$$^{**}$$	0.753$$^{***}$$	0.791$$^{***}$$	10.000$$^{*}$$
	(0.521)	–	–	(3.082)	(0.067)	(0.062)	(0.071)	(4.661)
IN	0.834$$^{**}$$	–	–	0.833	0.099$$^{*}$$	0.891$$^{***}$$	0.783$$^{***}$$	10.000$$^{*}$$
	(0.339)	–	–	(0.809)	(0.043)	(0.046)	(0.076)	(4.062)
CN	0.071	–	–	3.935$$^{*}$$	0.208$$^{**}$$	0.737$$^{***}$$	–	–
	(0.373)	–	–	(1.781)	(0.070)	(0.066)	–	–
ZA	1.006$$^{***}$$	–	–	1.625	0.325$$^{***}$$	0.666$$^{***}$$	–	–
	(0.237)	–	–	(1.012)	(0.097)	(0.091)	–	–

$$*$$, $$**$$, *and* $$***$$ *denote significance level at* $$5\%$$, $$1\%$$ *and* $$0,1\%$$, *respectively*

The model fit is evaluated in two ways. First, we check the model assumptions by comparing the sample quantiles of the standardized residuals with the theoretical quantiles. Figure shows the QQ-plot for all three oil price shocks and Fig. 5 for the stock market returns. They illustrate a good model fit with few deviations from the theoretical quantiles in the tails. Second, we performed the following goodness-of-fit tests: Kolmogorov-Smirnov, Cramer-von-Mises, Anderson Darling, and Neyman smooth test. None of the tests rejects the null hypothesis for any of the series. Consequently, we can use the estimated parametric distributions of the standardized residuals to perform the probability integral and obtain copula data for the further analysis, i.e.,

$$\begin{aligned} u_{i,t} = F_{i}\left( \frac{r_{it}-\hat{\mu }_{it}}{\hat{\sigma }_{it}}; \hat{\delta }_i|\mathcal {F}_{t-1}\right) , \end{aligned}$$

where $\hat{\mu }_{it} = E(r_{it}| \mathcal {F}_{t-1})$ and $\hat{\sigma }^2_{it} = Var(r_{it}| \mathcal {F}_{t-1})$ are the estimated conditional mean and standard deviation of the variable *i* at time *t* from the ARMA-GARCH model, and $F( \cdot ; \hat{\delta }_i)$ is the distribution function of the standardized residuals with estimated parameter vector $\hat{\delta }_i$.

**Fig. 4:**
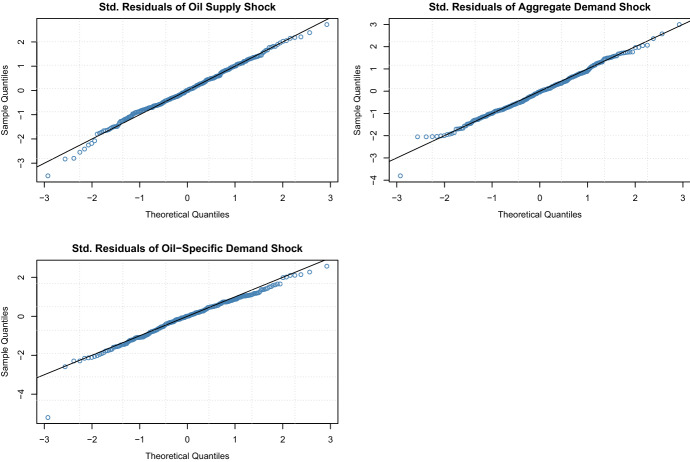
QQ-plot of the standardized residuals from the ARMA-GARCH models for the oil price shocks

**Fig. 5:**
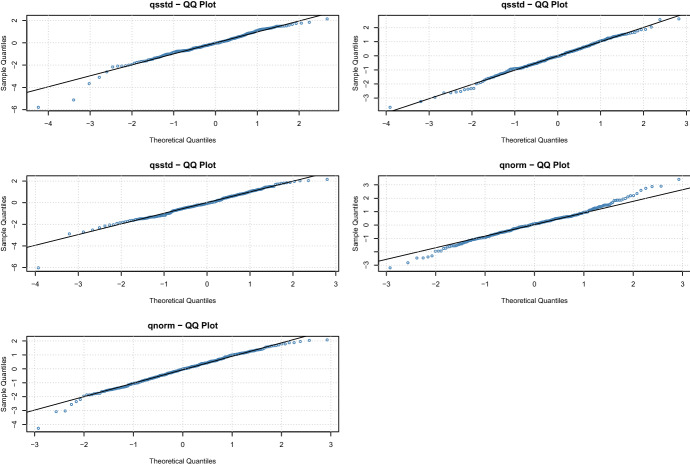
QQ-plot of the standardized residuals from the ARMA-GARCH models for the log returns of the BRICs countries

### Bivariate analysis

We begin by estimating appropriate dynamic copula models for each stock market with the three oil price shock variables, respectively.

For modeling the dynamics in dependence over time, the GAS copula model is used.^5^ The estimation of the best dynamic GAS copula is done with maximum likelihood estimation, where we allow for the following copula families: Gaussian, Student t, Clayton, Gumbel, Survival Clayton, and Survival Gumbel. The selection of the best copula family is based on the Akaike Information Criterion (AIC). In order to replicate the whole dependence spectrum (negative and positive dependence) for the Clayton, Gumbel, and their survival copulas, we allow the model to use the $90^{\circ }$-rotated versions of these copulas, whenever the dependence is negative.

**Table 4:** Summary statistics of dynamic Kendall’s Tau

	Copula family	Mean	Median	Max	Min	Sd	Pos.Prop.*
BR-SS	Surv. Clayton	−0.024	−0.021	0.306	−0.275	0.047	0.197
BR-DS	Gaussian	0.136	0.163	0.383	−0.136	0.100	0.886
BR-OS	Clayton	0.042	0.045	0.298	−0.339	0.056	0.855
RU-SS	Gumbel	0.041	0.033	0.164	−0.211	0.036	0.945
RU-DS	Student	0.062	0.062	0.222	−0.117	0.064	0.828
RU-OS	Gaussian	0.181	0.205	0.322	0.016	0.092	1.000
IN-SS	Surv. Clayton	0.004	0.005	0.371	−0.130	0.040	0.614
IN-DS	Gaussian	0.124	0.113	0.436	−0.143	0.121	0.869
IN-OS	Clayton	0.053	0.058	0.281	−0.106	0.042	0.907
CN-SS	Gumbel	0.001	0.001	0.073	−0.436	0.034	0.583
CN-DS	Gaussian	0.070	0.055	0.684	−0.464	0.164	0.728
CN-OS	Gaussian	0.070	0.067	0.392	−0.364	0.095	0.793
ZA-SS	Gumbel	0.013	0.005	0.313	−0.313	0.090	0.603
ZA-DS	Clayton	0.004	0.004	0.171	−0.155	0.021	0.697
ZA-OS	Gaussian	0.126	0.136	0.295	−0.057	0.048	0.979

*Pos.Prop. means the proportion of positive dependence in the total dynamic dependence

Table 4 provides summary statistics for the time-varying dependence parameter for each pair.^6^ Looking at the best fitting copula families, for 7 out of 15 pairs a symmetric copula was chosen, the Gauss copula in most cases. In terms of asymmetry, the Gumbel and survival Clayton copulas are characterized by upper tail dependence and lower tail independence, whereas for the Clayton copula we have lower tail dependence and upper tail independence. We can conclude that for all BRICS countries and the oil supply shocks, upper tail dependence is present (survival Clayton and Gumbel copulas). This means that when the supply of oil is extremely high, then also the stock market returns tend to be high, which is to be expected. For oil the exporters, i.e., Russia and Brazil, high oil supply has a direct effect on the profitability of companies in the oil export sector. For the oil-importing countries high oil supply is beneficial via lower prices. Further, we can conclude from the upper tail dependence between the aggregate demand shock and Russia’s stock market (Student copula) that an extremely high global demand level will result in rising stock returns. This is reasonable, because Russia is an oil-exporting country and its economy is highly dependent on oil companies. Moreover, for ZA-DS, RU-DS, BR-OS, and IN-OS we have evidence for lower tail dependence. Turning to the strength of dependence measured in terms of Kendall’s tau, while the average dependence is close to zero, there is some notable variation. While overall positive dependence dominates, there are also periods with considerable negative dependence.

**Table 5:** Summary statistics of $CoVaR-VaR$

	${CoVaR^{D}_{\alpha } - VaR^D_\alpha }$				${CoVaR^{U}_{\alpha } - VaR^U_\alpha }$			
	Mean	Max	Min	Sd	Mean	Max	Min	Sd
BR-SS	0.670	9.101	−3.820	1.133	−0.324	4.660	−4.480	0.954
BR-DS	−3.283	8.229	−7.882	2.860	1.749	4.201	−4.614	1.546
BR-OS	−2.623	12.111	−11.659	2.626	0.160	2.263	−10.495	0.811
RU-SS	−1.098	7.973	−11.973	1.502	1.967	16.507	−5.243	1.961
RU-DS	−4.587	1.760	−22.647	3.172	2.952	14.548	−1.151	2.037
RU-OS	−5.789	−0.984	−21.230	3.135	3.712	13.565	0.640	2.001
IN-SS	0.065	3.618	−4.106	0.584	0.221	5.844	−2.595	0.801
IN-DS	−2.193	3.759	−8.446	2.074	1.376	5.283	−2.450	1.309
IN-OS	−2.477	2.621	−10.909	1.835	0.269	2.769	−1.394	0.337
CN-SS	0.052	21.157	−1.254	1.297	0.158	3.166	−16.676	1.195
CN-DS	−0.976	10.281	−9.813	2.976	0.976	9.813	−10.281	2.976
CN-OS	−1.127	12.091	−10.626	2.143	1.127	10.626	−12.091	2.143
ZA-SS	0.005	12.385	−4.685	1.280	0.308	6.285	−11.981	1.592
ZA-DS	−0.148	8.923	−3.692	0.728	−0.030	0.997	−8.512	0.523
ZA-OS	−1.491	1.481	−7.626	0.963	1.491	7.626	−1.481	0.963

Based on the estimated models, the *VaR* and *CoVaR* are computed as explained in Sect. 2.4 using $\alpha =\beta =0.05$. We want to test for (1) the existence and the significance of risk spillover effects and (2) asymmetric effects of CoVaRs. This is done by comparing the upside and the downside *CoVaR* and *VaR* and testing their equality. Table 5 provides summary statistics for the differences between the *CoVaR* and the *VaR*. While the *CoVaR* tends to exceed the *VaR* in absolute terms there is a notable variation presents. To additionally assess the asymmetric effects, we construct two additional samples, namely $\frac{CoVaR^D_{\alpha }}{VaR^D_{\alpha }}$ and $\frac{CoVaR^U_{\alpha }}{VaR^U_{\alpha }}$, and test their equality. We test these hypotheses using the two-sample Kolmogorov–Smirnov (KS) test:

$$\begin{aligned} D_{n,m} = \sup _{x} |F_{1,n}(x)-F_{2,m}(x)|, \end{aligned}$$

where $F_{1,n}$ and $F_{2,m}$ are the empirical distribution functions of the first and the second sample, respectively.

Table 6 summarizes the results of the KS test for risk spillover and asymmetric effects. There are no significant risk spillover effects from oil supply shocks to BRICS stock returns, except in Brazil (upside and downside) and Russia (upside), the oil-exporting countries in the sample. However, there are significant risk spillover effects from aggregate demand oil shocks to BRICS stock returns, except in South Africa (upside and downside). Furthermore, there are significant risk spillover effects from oil-specific demand shocks to almost all BRICS stock returns, except in Brazil (upside) and in India (upside). In addition, we get significant results for asymmetric effects between upside and downside risk spillovers of almost all oil price shocks on all log returns of the BRICS countries, with an exception of China and the oil demand shocks and the oil-specific shocks.

**Table 6:** Kolmogorov–Smirnov test for spillover effect and asymmetry

	$H_0$: $CoVaR^D_{\alpha } = VaR^D_{\alpha }$	$H_0$: $CoVaR^U_{\alpha } = VaR^U_{\alpha }$	$H_0$: $\frac{CoVaR^D_{\alpha }}{VaR^D_{\alpha }} = \frac{CoVaR^U_{\alpha }}{VaR^U_{\alpha }}$
	$H_1$: $CoVaR^D_{\alpha } \ne VaR^D_{\alpha }$	$H_1$: $CoVaR^U_{\alpha } \ne VaR^U_{\alpha }$	$H_1$: $\frac{CoVaR^D_{\alpha }}{VaR^D_{\alpha }} \ne \frac{CoVaR^U_{\alpha }}{VaR^U_{\alpha }}$
BR,SS	$$0.183^{***}$$	$$0.162^{***}$$	$$0.138^{***}$$
BR,DS	$$0.566^{***}$$	$$0.507^{***}$$	$$0.548^{***}$$
BR,OS	$$0.397^{***}$$	0.076	$$0.797^{***}$$
RU,SS	0.079	$$0.152^{***}$$	$$0.493^{***}$$
RU,DS	$$0.252^{***}$$	$$0.217^{***}$$	$$0.441^{***}$$
RU,OS	$$0.400^{***}$$	$$0.341^{***}$$	$$0.548^{***}$$
IN,SS	0.045	0.062	$$0.372^{***}$$
IN,DS	$$0.259^{***}$$	$$0.266^{***}$$	$$0.269^{***}$$
IN,OS	$$0.331^{***}$$	0.076	$$0.848^{***}$$
CN,SS	0.021	0.048	$$0.221^{***}$$
CN,DS	$0.110^{*}$	$0.110^{*}$	0.021
CN,OS	$$0.155^{***}$$	$$0.155^{***}$$	0.021
ZA,SS	0.031	0.062	$$0.128^{**}$$
ZA,DS	0.045	0.021	$$0.541^{***}$$
ZA,OS	$$0.210^{***}$$	$$0.210^{***}$$	$$0.476^{***}$$

$$*$$, $$**$$ *and* $$***$$ *denote significance level at* $$10\%$$, $$5\%$$ *and* $$1\%$$, *respectively*

### Multivariate analysis

In this section we want to investigate multivariate risk spillover effects by combining several variables in our analysis. Again, we measure the risk spillover effects by estimating the upside and downside value at risks (VaRs) and conditional value at risks (CoVaRs). We calculate the VaR at a 95% confidence level ($\alpha = 0.05$). The CoVaR is defined as the VaR for BRICS stock returns at a 95% confidence level ($\alpha = 0.05$), conditional on the VaR for several variables at the 95% confidence level ($\beta = 0.05$). For calculating the multivariate CoVaRs, we combine the GAS model from Sect. 2.1 and the flexible D-vine-based quantile regression model from Sect. 2.5. Using this approach, we can analyze the effects on a country when all oil price components are shocked. Moreover, we are able to answer the question, how a stock market in one specific country would react to several oil price shocks and/or extreme stock market situation in other BRICS countries.

Let us say a few words on the D-vine copula model, estimated for the D-vine-based quantile regression. Due to the high dimension and complexity of our model and the increasing estimation uncertainties in the higher D-vine trees, we decide to allow for time constant copulas, in order to minimize the number of parameters to be estimated. We rely on the AIC for selecting the best pair copula. That means that for each pair copula in the D-vine copula model we estimate time-varying and constant copulas from the following families: Normal, Student t, Clayton, Survival Clayton, Gumbel, Survival Gumbel, and Independence copula.

Based on the D-vine estimation results, we compute the downside (upside) CoVaR of the variable of interest, say *i*, conditional on, e.g., OS, DS, SS being simultaneously equal to their downside (upside) VaR. We can calculate this by using Eq. (2.12) and the estimated conditional mean and standard deviation from the ARMA-GARCH model:

3.3 $$\begin{aligned} CoVaR_{\alpha ,t}^{D,i|OS,DS,SS} =&\hat{\sigma }_{i,t}\cdot \hat{q}_{\alpha ,i}(\hat{F}_{OS}^{-1}(\beta ), \hat{F}_{DS}^{-1}(\beta ), \hat{F}_{SS}^{-1}(\beta ))+\hat{\mu }_{i,t} \end{aligned}$$

3.4 $$\begin{aligned} CoVaR_{\alpha ,t}^{U,i|OS,DS,SS} =&\hat{\sigma }_{i,t}\cdot \hat{q}_{1-\alpha ,i}(\hat{F}_{OS}^{-1}(1-\beta ), \hat{F}_{DS}^{-1}(1-\beta ), \hat{F}_{SS}^{-1}(1-\beta ))+\hat{\mu }_{i,t}. \end{aligned}$$

We consider two types of analyses. First, we consider each countries stock market return conditional on all three oil shocks. Second, we look at the CoVaR of each country conditional on the most influential oil prices shock together with a shock to either one or all other BRICS stock markets. This allows us to see how influential multiple shocks are on the estimated CoVaR relative to the univariate VaR and the bivariate CoVaR.

We do not present detailed estimation results for the D-vine copula models, but these details can be found in Kielmann (2020). Graphical representations of the VaR and CoVaR for all studied countries and different conditioning variables can be found there as well. Below, we put some focus on the presentation of the results for China given its importance in the global economy.

We use a Kolmogorov–Smirnov (KS) test to test whether the CoVaR of each countries stock market returns, conditional on all three oil price shocks, significantly differs from the CoVaR conditional on the oil-specific demand shocks (OS). To conduct the test, we use the two time series of the CoVaRs ($CoVaR_{i|OS}$ and $CoVaR_{i|OS,DS,SS}$) for the downside and for the upside risk effect for country *i*, respectively. To assess the (conditionally) asymmetric effects, we construct two additional samples, $\frac{CoVaR^{i|OS,DS,SS}_{\alpha , D}}{CoVaR^{i|OS}_{\alpha ,D}}$ and $\frac{CoVaR^{i|OS,DS,SS}_{\alpha , U}}{CoVaR^{i|OS}_{\alpha ,U}}$, and test their equality. The tests reject all hypotheses with one exception, namely the upside CoVaR for Russia.^7^ Hence, we can conclude that inclusion of all oil price shocks in the risk spillover analysis leads to significant differences for the upside risk and for the downside risk, compared to the inclusion of only one oil price shock. Moreover, (conditionally) asymmetric effects cannot be rejected.

The summary statistics of the risk measures can be found in Tables 7 and 8. It can be seen that, as to be expected, multiple oil prices shocks lead to an increase in the risk measure. Looking at the different oil shocks in Table 7, we can see that supply shocks (SS) do not increase the risk, whereas demand (DS) and oil prices-specific (OS) shock do increase the risk notably, especially for the oil exporters Russia and Brazil. It is notable that joint shocks of all three oil price components increase the risk significantly compared to shocks in the individual components. This is not surprising as, by construction, the oil shocks are (unconditionally) orthogonal to each other. The largest increase in risk due to oil price shocks can be observed for Russia, which is not surprising given the dependence of the Russian economy on oil.

Table 8 shows the results for the CoVaR conditional on (1) the most influential oil shock alone, (2) together with the most influential stock market shock, (3) together with a shock to all BRICS stock markets, and (4) together with country-specific shocks to inflation and exchange rates (vs. the US$) as proxies for macroeconomic shocks. As macroeconomic shocks we consider devaluations of the exchange rate and high inflation, which are typically seen as negative macroeconomic conditions.^8^ This is done as, obviously, spillovers will occur between international stock markets and we want to evaluate how strong these spillovers are. Furthermore, macroeconomic shocks are also considerable risk factors and we want to compare their effect to shocks to the other risk factors. As to be expected, a larger number of shocks increases the CoVaR with the exception of China and South Africa, for which the risk conditional on shocks to all BRICS countries is not larger than conditionally only on the country with the strongest dependence. Comparing Tables 7 and 8, we can conclude that China and South Africa are less vulnerable to BRICS stock market shocks compared to the other countries after controlling for the most relevant oil shocks. Furthermore, for Brazil, Russia, India, and South Africa macroeconomic shocks increase the CoVaR risk in a similar magnitude as oil or stock market shocks. For China shocks to inflation and exchange rates have no noticeable spillover effects.

**Table 7:** CoVaR and VaR conditional on oil price shocks

	Downside				Upside			
	Mean	Max	Min	Sd	Mean	Max	Min	Sd
$\mathbf {VaR_{BR}}$	−13.664	−9.567	−27.520	3.567	12.307	23.196	9.087	2.803
$\mathbf {CoVaR_{BR|SS}}$	−12.001	−8.372	−24.272	3.159	11.605	21.825	8.583	2.631
$\mathbf {CoVaR_{BR|DS}}$	−16.947	−11.834	−25.848	3.039	14.056	21.271	10.823	2.293
$\mathbf {CoVaR_{BR|OS}}$	−16.287	−6.494	−38.476	4.933	12.467	23.941	5.695	2.919
$\mathbf {CoVaR_{BR|OS,DS,SS}}$	−17.488	−12.313	−34.988	4.505	14.361	27.207	10.563	3.307
$\mathbf {VaR_{RU}}$	−19.767	−9.431	−75.235	10.703	18.442	65.042	9.758	8.992
$\mathbf {CoVaR_{RU|SS}}$	−19.767	−9.431	−75.235	10.703	18.442	65.042	9.758	8.992
$\mathbf {CoVaR_{RU|DS}}$	−26.253	−12.688	−99.048	14.046	19.226	67.922	10.152	9.396
$\mathbf {CoVaR_{RU|OS}}$	−25.556	−13.342	−91.994	12.431	22.154	75.841	12.651	10.063
$\mathbf {CoVaR_{RU|OS,DS,SS}}$	−32.788	−17.589	−118.653	16.052	23.039	79.114	13.175	10.512
$\mathbf {VaR_{IN}}$	−11.088	−6.226	−21.990	3.228	10.776	19.867	6.722	2.692
$\mathbf {CoVaR_{IN|SS}}$	−12.298	−6.942	−24.306	3.556	11.545	21.338	7.177	2.900
$\mathbf {CoVaR_{IN|DS}}$	−13.281	−7.525	−25.393	3.437	12.152	21.002	7.696	2.729
$\mathbf {CoVaR_{IN|OS}}$	−13.696	−7.770	−26.982	3.934	11.39	21.042	7.085	2.858
$\mathbf {CoVaR_{IN|DS,OS,SS}}$	−17.440	−6.433	−42.743	5.756	12.877	28.225	5.750	3.464
$\mathbf {VaR_{CN}}$	−12.394	−6.828	−26.892	4.115	12.536	27.035	6.971	4.115
$\mathbf {CoVaR_{CN|SS}}$	−12.394	−6.828	−26.892	4.115	12.536	27.035	6.971	4.115
$\mathbf {CoVaR_{CN|DS}}$	−13.370	−0.831	−33.506	5.779	13.512	33.649	0.974	5.779
$\mathbf {CoVaR_{CN|OS}}$	−13.520	−5.115	−37.518	4.933	13.663	37.661	5.257	4.933
$\mathbf {CoVaR_{CN|OS,DS,SS}}$	−16.424	−2.000	−44.400	7.151	15.382	42.723	0.805	6.674
$\mathbf {VaR_{ZA}}$	−7.521	−3.333	−34.789	3.788	9.533	36.800	5.345	3.788
$\mathbf {CoVaR_{ZA|SS}}$	−7.517	−2.713	−28.566	3.693	9.841	33.984	5.219	3.915
$\mathbf {CoVaR_{ZA|DS}}$	−7.521	−3.333	−34.789	3.788	9.533	36.800	5.345	3.788
$\mathbf {CoVaR_{ZA|OS}}$	−9.153	−4.163	−41.639	4.513	11.165	43.651	6.175	4.513
$\mathbf {CoVaR_{ZA|OS,DS,SS}}$	−10.377	−4.786	−46.777	5.057	12.346	48.608	6.776	5.038

*This table presents the VaR and CoVaR conditional on the three types of oil price shocks defined in Sect. *3.1: *Supply shocks *(*SS*), *aggregate demand shocks *(*DS*) *and oil-specific demand shocks (OS)*

**Table 8:** CoVaR and VaR conditional on oil price, stock market, and macroeconomic shocks

	Downside				Upside			
	Mean	Max	Min	Sd	Mean	Max	Min	Sd
$\mathbf {VaR_{BR}}$	−13.664	−9.567	−27.520	3.567	12.307	23.196	9.087	2.803
$\mathbf {CoVaR_{BR|OS}}$	−16.287	−6.494	−38.476	4.933	12.467	23.941	5.695	2.919
$\mathbf {CoVaR_{BR|RU,OS}}$	−22.490	−15.709	−45.183	6.003	17.083	32.583	12.467	4.079
$\mathbf {CoVaR_{BR|BRICS,OS}}$	−25.450	−17.982	−49.568	6.416	18.953	35.553	13.982	4.341
$\mathbf {CoVaR_{BR|OS,ER,Infl.}}$	−20.974	−14.817	−41.795	5.360	16.189	30.777	11.875	3.756
$\mathbf {VaR_{RU}}$	−19.767	−9.431	−75.235	10.703	18.442	65.042	9.758	8.992
$\mathbf {CoVaR_{RU|OS}}$	−25.556	−13.342	−91.994	12.431	22.154	75.841	12.651	10.063
$\mathbf {CoVaR_{RU|BR,OS}}$	−31.684	−13.073	−121.079	16.516	28.523	106.288	12.064	13.792
$\mathbf {CoVaR_{RU|BRICS,OS}}$	−34.892	−16.631	−128.536	18.265	28.173	99.955	14.511	13.826
$\mathbf {CoVaR_{RU|OS,ER, Infl.}}$	−31.117	−15.131	−116.906	16.553	23.966	85.324	12.532	11.839
$\mathbf {VaR_{IN}}$	−11.088	−6.226	−21.990	3.228	10.776	19.867	6.722	2.692
$\mathbf {CoVaR_{IN|DS}}$	−13.281	−7.525	−25.393	3.437	12.152	21.002	7.696	2.729
$\mathbf {CoVaR_{IN|ZA,DS}}$	−18.000	−3.446	−36.500	6.427	15.248	28.968	5.883	4.549
$\mathbf {CoVaR_{IN|BRICS,DS}}$	−19.653	−10.861	−39.458	5.621	16.057	29.980	9.838	4.121
$\mathbf {CoVaR_{IN|DS,ER, Infl.}}$	−20.212	−11.629	−39.456	5.698	14.450	26.900	8.898	3.686
$\mathbf {VaR_{CN}}$	−12.394	−6.828	−26.892	4.115	12.536	27.035	6.971	4.115
$\mathbf {CoVaR_{CN|OS}}$	−13.520	−5.115	−37.518	4.933	13.663	37.661	5.257	4.933
$\mathbf {CoVaR_{CN|BR,OS}}$	−16.630	−9.173	−36.056	5.513	13.722	29.599	7.627	4.506
$\mathbf {CoVaR_{CN|BRICS,OS}}$	−16.884	−9.404	−36.758	5.565	14.015	30.903	7.802	4.623
$\mathbf {CoVaR_{CN|OS,ER, Infl.}}$	−12.394	−6.828	−26.892	4.115	12.536	27.035	6.971	4.115
$\mathbf {VaR_{ZA}}$	−7.521	−3.333	−34.789	3.788	9.533	36.800	5.345	3.788
$\mathbf {CoVaR_{ZA|OS}}$	−9.153	−4.163	−41.639	4.513	11.165	43.651	6.175	4.513
$\mathbf {CoVaR_{ZA|BR,OS}}$	−11.678	−4.461	−54.282	5.981	13.882	56.364	6.471	6.140
$\mathbf {CoVaR_{ZA|BRICS,OS}}$	−10.737	−1.798	−49.046	5.468	12.851	51.828	4.425	5.544
$\mathbf {CoVaR_{ZA|OS,ER, Infl.}}$	−9.153	−4.163	−41.639	4.513	11.165	43.651	6.175	4.513

* This table presents the VaR and CoVaR conditional on the single most important oil shock for each country, respectively, together with a shock to the stock market with the strongest dependence, together with a shock to all BRICS stock markets, and together with a shock to the exchange rate and the inflation of the respective country*

**Fig. 6:**
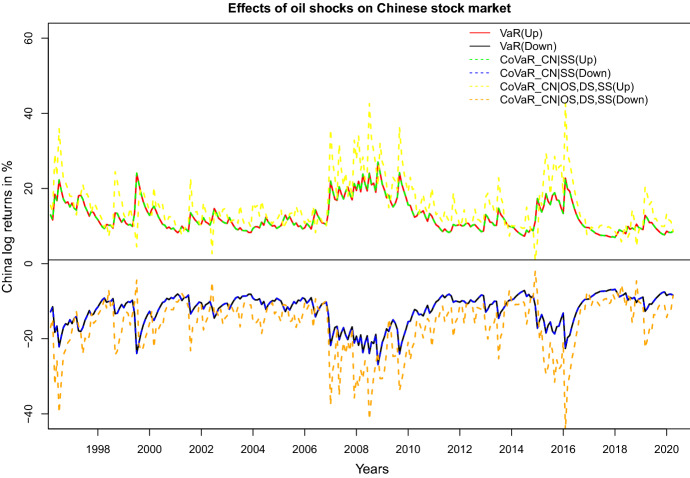
Upside and downside VaR and CoVaR of log returns in China conditional on specified oil shocks

Focusing on the example of China^9^, Fig. 6 depicts the evolution of the downside resp. upside VaR and CoVaR of the Chinese stock market returns, conditional on all oil shocks being equal to their VaR. For comparison, we also present the CoVaR conditional on only the oil-specific shocks being equal to its VaR. Specific crisis periods are clearly visible from the figure. For the most time points in the observed time period, we see that both the downside and the upside CoVaRs from the model with all oil shocks are higher than the CoVaRs from the model with only the oil-specific demand shocks. However, we observe that at some time points, the CoVaR conditional on all three oil price shocks being equal to their VaR is even lower than the VaR. This can be explained by the time-varying dependence permitted in our model, which identifies periods of negative dependence.

**Fig. 7:**
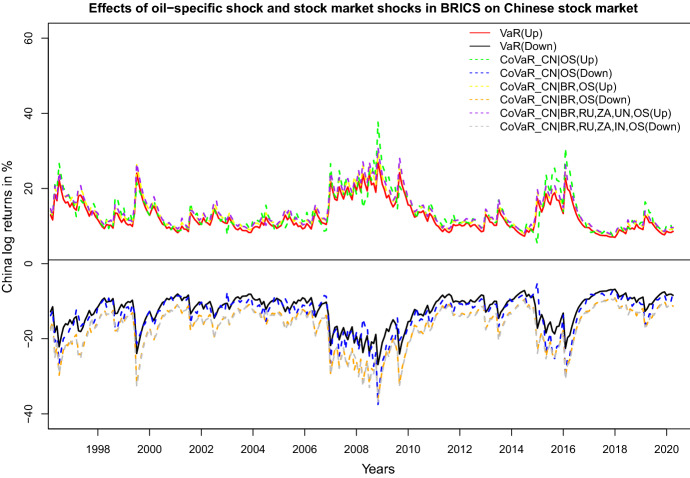
Upside and downside VaR and CoVaR for China conditional on specified oil and stock market shocks

In order to analyze the risk spillover effects from oil-specific shocks (OS) jointly with shocks in the stock markets in BRICS being equal to their VaR on the Chinese stock market, we apply the D-vine model with the ordering CN-BR-RU-ZA-IN-OS. Figure 7 depicts the CoVaRs for the model with all BRICS and the oil-specific demand shocks, for the model with Brazil and the oil-specific demand shocks, and for the model with only the oil-specific demand shocks. We observe that the extreme market risks in Chinese stock market does not change much if considering all BRICS stock market in our analysis, compared to our model if only including the Brazil stock market and the oil-specific shocks. From Figs. 6 and 7, we can see that the Covid-19 shock in early 2020 is not visible in the (conditional) risk level of the Chinese stock market.

## Conclusion

This paper quantifies risk spillover effects from different types of oil price shocks to the stock markets of the BRICS countries in a multivariate setting relying on three methodological pillars. First, we base our risk spillover analysis on the bivariate time-varying GAS model of Creal et al. (2008), a recommendable specification for introducing time variation in copula models. Second, we extend the bivariate copula-based analysis of a risk spillover to a multivariate one. Third, using the D-vine-based quantile regression of Kraus and Czado (2017), we are able to investigate the effects of several oil price shocks simultaneously on BRICS countries’ stock returns and, additionally, consider interdependencies between BRICS stock markets and with macroeconomic variables.

Our empirical results provide clear evidence that the dependence between oil shocks and stock returns is time-varying and mostly positive. However, the extent of this dependence strongly depends on the type of oil price shocks and the importance of oil for an individual country. In general, the oil-specific shocks are the most influential oil price shocks for BRICS stock market movements, whereas the oil supply shocks hardly influence the stock markets and their extreme market risk level. Furthermore, our analysis of risk spillover effects of extreme risks in the oil market shows us that especially demand oil shocks influence the risk level of stock returns in the BRICS.

We observe that the Asian financial crisis, the global financial crisis, and the Covid-19 crisis led to significantly increasing risks in the stock markets. However, we also found that China’s stock market has recovered quickly from the Covid-19 crisis. Based on our multivariate risk spillover analysis, we can conclude that including all oil price shocks in our analysis, instead of only one oil price shock, leads to higher risk spillover effects on the stock markets in BRICS. Moreover, we find that there are considerable interdependencies between the BRICS stock markets leading to considerable risk spillovers in addition to oil shocks. Finally, joint shocks to inflation and exchange rates also spill over to the respective stock markets for Russia, Brazil, and South Africa, whereas they do not for China and India.

The described empirical findings provide important implications for financial investors to improve their portfolio strategies and risk positions. They are also are helpful for the governments in BRICS countries, in order to react better to oil price shocks or BRICS wide stock market shocks. From a policy perspective, we saw that stock markets of oil-exporting countries are more vulnerable to oil demand shocks. Reducing this vulnerability is crucial for preventing significant economic and financial instability in these countries that may spill over to other countries via financial and non-financial contagion channels.

The methodology we suggest should be useful in different applications. The D-vine-GAS copula model is highly flexible and can be applied in large dimensions without exploding computational requirements. Combined with the D-vine-based quantile regression this allows for a flexible and general risk spillover analysis compared to the commonly used bivariate analysis in which the CoVaR is computed based on a single risk event. Conditioning on multiple joint risk events improves the empirical usefulness of the this risk measure. This methodological contribution could be of interest in a number of applications as it increases the usefulness for copula-based CoVaR estimation.

## Footnotes

1 If the parameter is vector valued, the score is a vector to be scaled by the square root of the information matrix.

2 Multivariate density forecasts could, e.g., be evaluated using the method studied in Dovern and Manner (2020).

3 Although in principle one can consider the VaR of a given portfolio.

4 https://sites.google.com/site/lkilian2019/.

5 As an alternative, the time-varying model by Patton (2006) was used, but the model fit was clearly inferior in comparison with the GAS model.

6 Detailed results for the bivariate analysis can be found in Kielmann (2020).

7 Detailed results for these hypothesis tests can be found in Kielmann (2020).

8 For the macroeconomic data we consider standardized residuals from the best-fitting ARMA models with ARCH(1) errors, as there is only weak evidence for ARCH effects in these series. Details concerning the modeling of these series are omitted to preserve space and can be obtained by the authors upon request.

9 Similar graphs and more detailed results for the other countries are available from the authors upon request.
